# The generalisability of randomised clinical trials: an interim external validity analysis of the ongoing SENOMAC trial in sentinel lymph node-positive breast cancer

**DOI:** 10.1007/s10549-020-05537-1

**Published:** 2020-01-27

**Authors:** Jana de Boniface, Johan Ahlgren, Yvette Andersson, Leif Bergkvist, Jan Frisell, Dan Lundstedt, Roger Olofsson Bagge, Lisa Rydén, Malin Sund, the SENOMAC Trialists’ Group, Peer Christiansen, Tove Filtenborg Tvedskov, Birgitte Vrou Offersen, Toralf Reimer, Thorsten Kühn, Michalis Kontos, Oreste Gentilini, Roland Reitsamer

**Affiliations:** 1 Department of Surgery, Capio St Göran’s Hospital, Stockholm, Sweden; 2 Department of Molecular Medicine and Surgery, Karolinska Institutet, Stockholm, Sweden; 3 Department of Oncology, University of Örebro, Örebro, Sweden; 4 Regional Oncologic Centre, Uppsala-Örebro Health Care Region, Uppsala, Sweden; 5 Department of Surgery, Västmanland County Hospital, Västerås, Sweden; 6 Västmanland County Hospital, Center for Clinical Research, Uppsala University, Västerås, Sweden; 7 Division of Cancer, Department of Breast, Endocrine Tumours and Sarcoma, Karolinska Universitety Hospital, Stockholm, Sweden; 8 Department of Oncology, Sahlgrenska University Hospital, Institute of Clinical Sciences, Sahlgrenska Academy, University of Gothenburg, Göteborg, Sweden; 9 Wallenberg Centre for Molecular and Translational Medicine, University of Gothenburg, Göteborg, Sweden; 10 Department of Surgery, Institute of Clinical Sciences, Sahlgrenska University Hospital, Sahlgrenska Academy at the University of Gothenburg, Göteborg, Sweden; 11 Division of Surgery, Department of Clinical Sciences Lund, Lund University, Lund, Sweden; 12 Department of Surgery and Gastroenterology, Skåne University Hospital, Lund, Sweden; 13 Surgery Center, Norrland University Hospital, Umeå, Sweden; 14 Department of Surgical and Perioperative Science/Surgery, Umeå University, Umeå, Sweden

**Keywords:** Breast cancer, Omission of axillary dissection, Clinical trial, Sentinel lymph node biopsy

## Abstract

**Purpose:**

None of the key randomised trials on the omission of axillary lymph node dissection (ALND) in sentinel lymph-positive breast cancer have reported external validity, even though results indicate selection bias. Our aim was to assess the external validity of the ongoing randomised SENOMAC trial by comparing characteristics of Swedish SENOMAC trial participants with non-included eligible patients registered in the Swedish National Breast Cancer Register (NKBC).

**Methods:**

In the ongoing non-inferiority European SENOMAC trial, clinically node-negative cT1–T3 breast cancer patients with up to two sentinel lymph node macrometastases are randomised to undergo completion ALND or not. Both breast-conserving surgery and mastectomy are eligible interventions. Data from NKBC were extracted for the years 2016 and 2017, and patient and tumour characteristics compared with Swedish trial participants from the same years.

**Results:**

Overall, 306 NKBC cases from non-participating and 847 NKBC cases from participating sites (excluding SENOMAC participants) were compared with 463 SENOMAC trial participants. Patients belonging to the middle age groups (*p* = 0.015), with smaller tumours (*p* = 0.013) treated by breast-conserving therapy (50.3 versus 47.1 versus 65.2%, *p* < 0.001) and less nodal tumour burden (only 1 macrometastasis in 78.8 versus 79.9 versus 87.3%, *p* = 0.001) were over-represented in the trial population. Time trends indicated, however, that differences may be mitigated over time.

**Conclusions:**

This interim external validity analysis specifically addresses selection mechanisms during an ongoing trial, potentially increasing generalisability by the time full accrual is reached. Similar validity checks should be an integral part of prospective clinical trials.

Trial registration: NCT 02240472, retrospective registration date September 14, 2015 after trial initiation on January 31, 2015

## Background

In the last decade, several large randomised clinical trials (RCTs) de-escalating axillary treatment interventions in sentinel lymph node (SLN)-positive breast cancer have revolutionised the clinical management of limited axillary nodal metastases [1–4], and results from further clinical trials, both ongoing (INSEMA [5], SENOMAC [6], POSNOC [7]) and completed (SOUND [8]), are eagerly awaited. As discussed in an important contribution by Ford and Norrie in 2016, clinical trials have a tendency to strictly select the healthiest subjects, and the concept of pragmatic trials has been suggested in order to increase external validity [9]. Accordingly, trial participants should be comparable to clinical practice patients in both patient and tumour characteristics, and the standard treatment within and outside a trial should be the same. It is ultimately the clinical practice population to whom clinical trial results will be applied, and divergences in characteristics will negatively affect external validity of a trial, in the worst case jeopardising a successful and safe implementation of the trial’s results.

In specific, the ACOSOG Z0011 and IBCSG 23-01 trials substantially changed routine management of breast cancer patients with 1–2 sentinel lymph node metastases. The former trial, Z0011, included patients with tumours up to 5 cm in size and up to two nodal metastases (which could be either macro- or micrometastases) treated by breast-conserving surgery combined with whole-breast radiotherapy. While the results of this trial that closed prior to target accrual and thus did not reach statistical power show no survival disadvantage for patients undergoing SLN biopsy only [2, 10], the characteristics of the analysed population clearly represent a selection: While one would expect a rate of micrometastatic disease amongst all SLN-positive cases of about 14% as published from Swedish prospective data [11], it is an astonishing 37.5% and 44.8% in the respective groups. A similar observation is true for the IBCSG 23-01 trial, where only patients with micrometastatic SLNs were eligible: While the size of micrometastases can be ≤ 2 mm, the observed size of micrometastases was ≤ 1 mm in 69% and 70% of cases in the respective groups [1].

The ongoing SENOMAC trial is a multicentre European RCT aiming to extend the findings of IBCSG 23-01 and ACOSOG Z0011 to those breast cancer patients treated by mastectomy, and additionally including cT3 patients and those with extranodal extension, while in parallel validating previous trial results in breast-conserving therapy. As the SENOMAC trial was initiated in Sweden, where the majority of patients have been included thus far, the Swedish National Breast Cancer Register (NKBC) is an ideal source of comparative background data from all newly diagnosed Swedish breast cancer patients registered during the trial enrolment period. Here, we compare NKBC data with trial data in order to assess the external validity of the trial and assure that future trial outcomes will be sufficiently representative of clinical practice patients.

## Methods

The main aim of this analysis was to assess whether Swedish SENOMAC participants included into the trial in 2016 and 2017 were representative of the breast cancer population that was reported to the Swedish National Breast Cancer Register (NKBC) during the same time period, and fulfilled trial eligibility criteria. The reason for choosing these years was (i) that most participating Swedish sites had been initiated by the beginning of 2016, and (ii) that NKBC data for these years had been completed during spring of 2018. Even though further international sites had joined the trial by 2017, it was decided only to evaluate Swedish data due to the easy access of NKBC data.

Inclusion criteria for the SENOMAC trial are shown in Table 1. All cases randomised in the years 2016 and 2017 were included, irrespective if data reported had by then been independently monitored or not. Patients that had terminated their trial participation early due to withdrawal of consent, physician’s choice, or changed histopathological information were not included. In NKBC, data on all cases of primary breast cancer in both genders of all ages are included, and completeness reaches 96–99% [12] with a data validity of over 90% [13]. Each breast cancer generates one separate case in NKBC, but in the present analysis, NKBC bilateral cases could not be identified as data were anonymised before delivery.

**Table 1:** Inclusion and exclusion criteria according to the SENOMAC study protocol

Inclusion criteria	Primary invasive breast cancer of clinical stage T1–T3^a^ No palpable lymph node metastases prior to sentinel node biopsy Preoperative ultrasound of the axilla performed Macrometastasis in not more than two lymph nodes at sentinel node biopsy Written informed consent Age 18 years or older
Exclusion criteria	Regional metastases outside of the ipsilateral axilla Distant metastases Pregnancy Bilateral invasive breast cancer, if one side meets any exclusion criteria Medical contraindication for radiotherapy or systemic treatment Inability to absorb or understand the meaning of the study information; for example, through disability, inadequate language skills, or dementia Prior history of invasive breast cancer

^a^According to the TNM classification system, AJCC Cancer Staging Manual, Eighth Edition, 2017

In order to match the SENOMAC trial population, data from NKBC were extracted with the following selection criteria:

(i) Diagnosis of primary invasive breast cancer in 2016 or 2017
(ii) Patient age at least 18 years
(iii) No evidence of distant metastases
(iv) SLN detected
(v) SLN biopsy shows 1–2 macrometastases

**Fig. 1:**
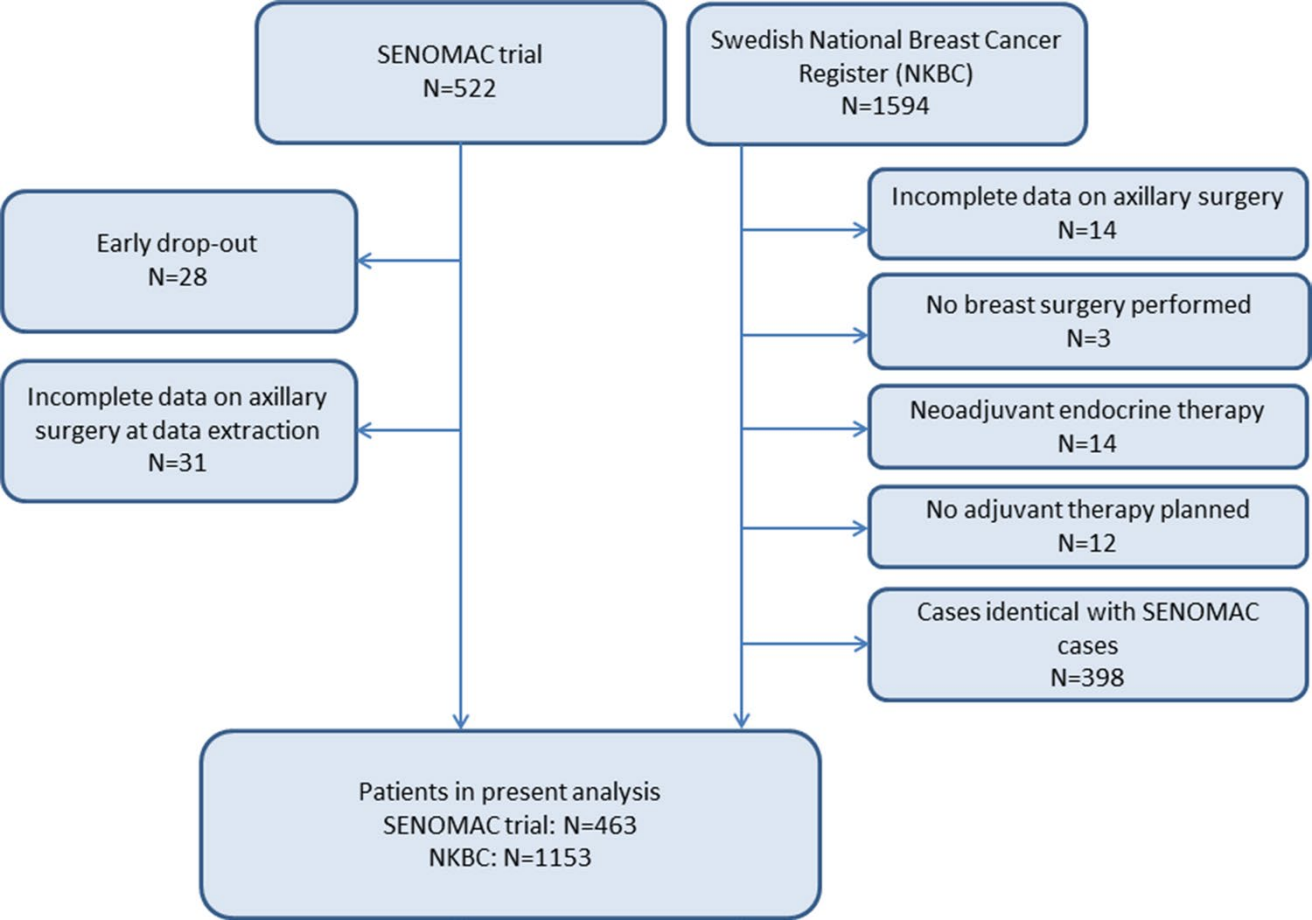
Flow diagram defining the original cohorts and the analysed sample

A number of trial criteria were not available from NKBC, such as the individuals’ ability to absorb the trial information and give informed consent, the performance of preoperative axillary ultrasound, palpability of axillary nodal metastases, pregnancy, and any contraindications to radiotherapy or systemic treatment. To meet the latter criterion, NKBC patients not being planned for any adjuvant therapy, not receiving any breast surgery, or planned for neoadjuvant endocrine therapy only were excluded (Fig. 1). At the beginning of 2016, 21 Swedish sites participated in the SENOMAC trial, three of which were combining two hospitals under the same administration. In the beginning of 2017, two more sites were activated and included in this analysis; for the assessment of inclusion rates per site, however, NKBC cases diagnosed at those two sites before their trial initiation date were not included.

In order to avoid overlapping of the SENOMAC and the NKBC cohorts—considering that trial participants are also registered in NKBC—we identified and excluded NKBC cases identical to SENOMAC participants by comparing the date of surgery at the same site, as well as age and finally tumour size. In case of doubt, tumour biology was also compared for certain identification of identical cases.

### Statistics

Descriptive data are presented as medians with their range (continuous data) or distributions with percentages (categorical data). For individuals treated with neoadjuvant chemotherapy (NACT), pre-treatment tumour biology based on core needle biopsy is reported. The same individuals were excluded from analyses of pathological tumour size, histopathological tumour type, and grade. Three cohorts were created: NKBC cases from sites not participating in SENOMAC, NKBC cases not included in SENOMAC but treated at participating sites, and SENOMAC participants. For comparison of the three cohorts, the Kruskal–Wallis test was applied for continuous data and the Chi-square or the Fisher’s exact test for categorical data. Exact two-sided significances are presented, and the level of significance was set at *p* < 0.05. For the calculation of the proportion of patients included in the SENOMAC trial per site, the number of participants per site was divided by the total reported number of eligible NKBC cases from the same time period. All analyses were performed using SPSS statistical software version 24 (IBM Corp., Armonk, NY, USA).

## Results

The SENOMAC trial cohort consisted of 522 individuals included by 23 Swedish sites; 28 early drop-outs had been registered during 2016 and 2017 (5.4%). Cases with incomplete information on performed axillary surgery at data extraction were excluded, leaving 463 individuals for analysis. The corresponding NKBC cohort initially comprised 1594 cases originating from all 40 Swedish hospitals reporting to NKBC. After the exclusion of the patient categories described previously, and the removal of NKBC cases registered both in the SENOMAC and the NKBC cohorts, 1153 cases remained in the analysis (see Fig. 1). In 66 trial participants, no identical NKBC case could be identified.

### Patient and tumour characteristics

Patient and tumour characteristics are presented in Table 2. Here, the three cohorts are defined as (i) NKBC patients operated at sites not participating in the SENOMAC trial, (ii) NKBC patients not included in the SENOMAC trial, even though operated at sites participating in the SENOMAC trial, and (iii) patients included in the SENOMAC trial.

**Table 2:** Patient and tumour characteristics in SENOMAC trial and NKBC populations

	NKBC Not participating sites *N* = 306	NKBC Participating sites excl. SENOMAC cases *N* = 847	SENOMAC trial *N* = 463	*p* Value
Patient age^b^	64 (32–94)	63 (26–95)	61 (30–88)	0.050*
Patient age group				0.015
< 41 years	13 (4.2)	62 (7.3)	16 (3.5)	
41–50 years	49 (16.0)	143 (16.9)	91 (19.7)	
51–65 years	102 (33.3)	262 (30.9)	169 (36.5)	
> 65 years	142 (46.4)	380 (44.9)	187 (40.4)	
Neoadjuvant chemotherapy^a^				< 0.001
Yes	5 (1.6)	78 (9.2)	14 (3.0)	
No	301 (98.4)	769 (90.8)	449 (97.0)	
Missing	0	0	0	
Breast surgery				< 0.001
Breast-conserving	154 (50.3)	399 (47.1)	302 (65.2)	
Mastectomy	152 (49.7)	448 (52.9)	161 (34.8)	
Missing	0	0	0	
Tumour size (mm)^b,c^	21 (5–100)	20 (1–150)	19 (1–125)	0.013*
Tumour stage^c^				0.035
pT1	145 (48.2)	397 (51.6)	265 (59.0)	
pT2	137 (45.5)	310 (40.3)	164 (36.5)	
pT3	16 (5.3)	50 (6.5)	20 (4.5)	
Missing	3 (1.0)	12 (1.6)	0	
Histological tumour type^c^				0.226
Ductal	226 (75.1)	623 (81.0)	358 (79.7)	
Lobular	61 (20.3)	114 (14.8)	75 (16.7)	
Other	14 (4.7)	27 (3.5)	16 (3.6)	
Missing	0	5 (0.7)	0	
Tumour multifocality				0.097
Multifocal	94 (30.7)	207 (24.4)	127 (27.4)	
Unifocal	205 (67.0)	620 (73.2)	336 (72.6)	
Missing	7 (2.3)	20 (2.4)	0	
Tumour histological grade^c^				0.075
1	50 (16.6)	92 (12.0)	66 (14.7)	
2	161 (53.5)	423 (55.0)	264 (58.8)	
3	89 (29.6)	249 (32.4)	118 (26.3)	
Missing	1 (0.3)	5 (0.7)	1 (0.2)	
Oestrogen receptor status^d^				0.010
Positive	278 (90.8)	736 (86.9)	437 (94.4)	
Negative	25 (8.2)	87 (10.3)	26 (5.6)	
Missing	3 (1.0)	24 (2.8)	0	
Progesterone receptor status^d^				0.214
Positive	230 (75.2)	630 (74.4)	375 (81.0)	
Negative	73 (23.9)	179 (21.1)	88 (19.0)	
Missing	3 (1.0)	38 (4.5)	0	
HER2 status^d^				0.137
Positive	40 (13.1)	106 (12.5)	43 (9.3)	
Negative	265 (86.6)	728 (86.0)	420 (90.7)	
Missing	1 (0.3)	13 (1.5)	0	
Proliferation (Ki67, %)^b,d^	20 (1–95)	25 (1–100)	23 (2–90)	< 0.001*

*NKBC*: Swedish National Breast Cancer Register

*p values are based on Kruskal–Wallis and Chi-square tests, respectively, comparing all three groups

^a^Patients receiving neoadjuvant chemotherapy (NACT), and undergoing sentinel lymph node biopsy before start of NACT, could only be included into the SENOMAC trial from February 22, 2016

^b^Median (range)

^c^Excluding patients receiving neoadjuvant systemic treatment;

^d^Based on core needle biopsy in patients receiving neoadjuvant systemic treatment and on surgical specimen in all other cases

There was a significant overrepresentation of the middle age groups in the trial. Median age showed a tendency to be lower in trial participants, but this was based on the fact that non-participating sites generally reported a significantly higher median patient age than participating sites (*p* = 0.048). When looking at time trends, age group differences were mitigated in the year 2017 and lost statistical significance even though the same pattern in age distribution persisted (*p* = 0.159).

NACT was an exclusion criterion to the SENOMAC trial up to February 22, 2016, and the fact that not all sites changed their enrolment patterns immediately after this protocol amendment probably explains why sites participating in the trial reported fewer NACT cases amongst trial participants. Accordingly, only 0.5% of trial participants had received NACT in 2016, a figure rising to 5% in 2017. The corresponding figures for the NKBC cohort were 3.3% and 5.5%. Still, the rate of NACT was generally higher at sites participating in the trial than at those not participating (7.0% versus 1.6%, *p* < 0.001). In order to assess whether the differences in ER negativity were associated with the late introduction of NACT cases into the trial, non-NACT patients treated at sites participating in the trial were selected; subsequently, those included in the trial were compared with those not included in the trial. Despite this, ER negativity was still significantly more common in not included patients (*p* = 0.026). Differences in proliferation index, however, lost their significance in this comparison (*p* = 0.094). In addition, sites not participating in the trial generally reported a lower proliferation index (Ki67 20%, range 1–95) than participating sites (25%, range 1–100; *p* < 0.001).

Median tumour size was lowest in the trial cohort, which also had the highest proportion of breast-conserving surgery. When comparing sites not participating in the trial with those participating, the mastectomy rate was still lower in the latter but without statistical significance (46.5% versus 49.7%, *p* = 0.340). When looking at the years 2016 and 2017, a time trend was seen for an increasing tumour size in those included in the SENOMAC trial from a median of 18 mm (range 1–125) to 20 mm (range 7–130). For the type of surgery, however, mastectomy rates in the trial cohort followed the same decreasing trend seen in the NKBC cohort, with declines from 36.6% to 31.1%, and from 49.9% to 43.0%, respectively.

### Axillary surgery

Results from axillary surgery are depicted in Table 3. Trial participants had a significantly higher proportion of only one macrometastases in their SLN biopsy, even though the total number of axillary metastases and excised lymph nodes did not differ. As this should most likely indicate a selection bias of lower risk patients into the trial, we investigated time trends, adding reported trial data from the year 2018. Here, the proportion of only one macrometastasis in the SLNB showed a steady decrease from 88.5% (2016) to 86.4% (2017) and 79.7% (2018).

**Table 3:** Axillary surgery results

	NKBC Not participating sites *N* = 306	NKBC Participating sites excl. SENOMAC cases *N* = 847	SENOMAC trial *N* = 463	*p* Value
Number of SLNs^a^	2 (1–8)	2 (1–9)	2 (1–7)	0.565*
Number of SLN macrometastases				0.001^#^
1	241 (78.8)	677 (79.9)	404 (87.3)	
2	65 (21.2)	170 (20.1)	59 (12.7)	
Number of excised LNs in total^a,b^	12 (2–36)	12 (2–49)	12 (1–34)	0.963*
Number of axillary metastases in total^a,b^	2 (1–19)	2 (1–24)	1 (1–27)	0.030*
Nodal stage^b^				
pN1	178 (80.5)	493 (84.0)	200 (89.3)	0.074^#^
pN2	37 (16.7)	72 (12.3)	19 (8.5)	
pN3	6 (2.7)	22 (3.7)	5 (2.2)	
Rate of non-SLN positivity (%)^b^	40.7	37.1	34.8	0.428^#^

*SLN* sentinel lymph node, *LN* lymph node

*Kruskal–Wallis test

^#^Chi-square test

^a^Median (range)

^b^Includes only those cases operated by axillary lymph node dissection

For the evaluation of non-SLN results, only those were selected who had undergone a completion axillary lymph node dissection (ALND). The incidence rates for non-SLN metastases did not differ between the cohorts, and was 34.8% in the SENOMAC trial. As dose-dense adjuvant chemotherapy may be considered for pN2-3 patients according to Swedish national guidelines, and such patients would not have been identified without an ALND, these were scrutinised in detail: Of 19 pN2 and 5 pN3 trial participants, being operated by ALND, one had received NACT. It needs to be underlined that pN stage in this latter case is a composite of pre-NACT SLN biopsy and post-NACT ALND, which was still the routine surgical sequence in Sweden at initiation of this analysis. Tumour characteristics, number of SLNs, and patient age in the 24 pN2-3 patients were not different from the reported values of the trial cohort in Table 2. Preoperative axillary ultrasound was performed in all cases, with no suspicious lymph nodes reported in 21 cases. In the remaining three cases with suspicious ultrasound findings, fine needle aspiration was negative in two. In the last case, a non-palpable nodal metastasis was verified by cytology, and the patient was enrolled into the trial according to protocol. The median size of the largest SLN metastasis was significantly larger in pN2-3 trial participants than in their pN1 counterparts (7 mm (3–32 mm) versus 5 mm (2–22 mm), *p* = 0.007), and a non-significant trend towards a higher proportion of extranodal extension was seen in pN2-3 patients (33.3% versus 22.5%, *p* = 0.106).

### Inclusion rates per site

In order to assess inclusion rates, the number of trial participants per participating site was divided by the number of eligible NKBC cases—according to those inclusion criteria available from NKBC—from the same sites during the same time period (Table 4). As two sites were only initiated in 2017, NKBC cases reported by those sites before the date of initiation were excluded. Overall, 463 out of 1311 NKBC cases registered at participating sites were enrolled in the trial (35.3%). It should be underlined that 66 trial participants could not be identified in the NKBC population, indicating that inclusion rates may be lower than presented here. Inclusion rates ranged between 7.3% and 63.0%, and there was no difference between Public University versus Public Non-University Hospitals, or high- versus low-volume hospitals, indicating the importance of a dedicated clinical trial team on site.

**Table 4:** Sites participating in the SENOMAC trial: proportion of included out of all eligible cases and presentation of site characteristics

Type of SENOMAC site^a^	Annual case load in 2017 (N)	Eligible cases in NKBC (N)	Included cases in SENOMAC (N)	Percentage in SENOMAC (%)
PU	431	106	44	41.5
PU	233	67	34	50.7
PU	202	54	14	25.9
PU	477	136	24	17.6
PU	161	46	16	34.8
PU^b^	413	109	32	29.4
PU	105	34	18	52.9
PNU	144	44	27	61.4
PNU	539	101	38	37.6
PNU	174	44	18	40.9
PNU	99	31	3	9.7
PNU^b^	178	54	34	63.0
PNU^b^	200	69	17	24.6
PNU	194	41	3	7.3
PNU	125	39	13	33.3
PNU	86	24	7	29.2
PNU	57	18	11	61.1
PNU	220	52	18	34.6
PNU	153	47	19	40.4
PNU	128	31	13	41.9
PNU^c^	194^a^	32	7	21.9
PNU^c^	158^a^	23	8	34.8
P	483	109	45	41.3
23	5154	1311	463	35.3

Numbers represent eligible and included cases in 2016 and 2017. Percentage in SENOMAC is the number of cases included in the SENOMAC Trial divided by the number of eligible cases registered in NKBC. NKBC: Swedish National Breast Cancer Register

^a^*PU* Public University Hospital, *PNU* Public Non-University Hospital, *P* Private Hospital

^b^Two hospitals constituting one site

^c^Site activated during 2017

## Discussion

This comparative analysis aimed to assess the external validity of the ongoing randomised SENOMAC trial by comparing patient and tumour characteristics of trial participants randomised in 2016 and 2017 with non-included eligible patients registered in the Swedish National Breast Cancer Register (NKBC) during the same time period. Some potential selection mechanisms could be identified: patients in middle age groups, with smaller tumours treated by breast-conserving therapy and less nodal tumour burden were over-represented. Time trends indicated, however, that differences decrease over time, thereby increasing the external validity of the trial population.

In order to increase external validity, and thus generalisability of RCT results, the concept of pragmatic trials, introduced already in 1967 [14], has recently gained increased attention [9, 15, 16]. Traditionally, one typical feature of pragmatic trials is the definition of wider inclusion criteria, allowing a broader and more representative patient population to enter the trial. In SENOMAC, inclusion criteria are well adapted to the general breast cancer population, and together with the remaining domains of the PRECIS-2 toolkit [17], this places the SENOMAC trial clearly towards the pragmatic end of the pragmatic/explanatory continuum. Despite this, generalisability may be threatened by selection mechanisms that impede the inclusion of the full range of potential participants into clinical trials: Clinicians may be reluctant to include high-risk patients into trials and thus expose them to potential hazards the trial intervention may seem to pose. On a higher level, this reluctance may preclude participation of sites that engage less in the support of progressive trials aiming at the de-escalation of surgical interventions. Consequently, high-impact clinical trials such as the previously cited ACOSOG Z0011 [2, 10] sometimes are flawed by a selection bias that negatively influences generalisability.

Our results showed a similar selection bias towards less advanced breast cancers, albeit this tendency seemed to decrease over time. One could speculate that this is due to treating physicians getting used to the de-escalated locoregional therapy in the experimental arm. If true, this may be due to impact from trial-related meetings and international publications, monthly letters from the trial committee, and discussions with colleagues. Since the validity check was performed while this trial still is ongoing, the opportunities to affect the selection bias increase. As reported previously, elderly patients are generally under-represented in clinical trials [18, 19]. This may well be due to restrictive inclusion criteria: In the case of SENOMAC, which is first and foremost a de-escalation trial, only patients able to receive adequate systemic treatment and/or radiotherapy were eligible. It is, however, important to underline that systemic treatment may well consist of only endocrine therapy in hormone receptor-positive cases, and that chemotherapy can hardly be seen as standard of care in the frail elderly. At the same time, elderly patients certainly do not have less potential benefit from a decrease of surgical complications and long-term morbidity than their younger counterparts. Also tumour size, and subsequently, rates of breast-conserving surgery were different in the trial population than the NKBC dataset. Given that previous trials such as ACOSOG Z0011 [2], IBCSG 23-01 [1], and AMAROS [3] included few or no mastectomy patients, the inclusion of such patients is still viewed as more controversial than the inclusion of patients receiving breast conservation: in mastectomy, tumours tend to be more advanced, and are, in contrast to breast conservation, not always treated by adjuvant radiotherapy. In addition, both individual surgeons and entire sites joining a de-escalation trial on axillary surgery may well be more prone to adapt a similarly progressive approach to breast surgery, yielding higher rates of breast conservation.

In SENOMAC, all Swedish hospitals treating breast cancer were invited to participate apart from six hospitals with annual caseloads of about 50 newly diagnosed breast cancer patients or less. Of all invited sites, only four still remain outside the trial today; these have annual caseloads of 138, 199, 200, and 269, respectively (as of year 2017), and are non-university hospitals. Thus, all types of hospitals are well represented in the trial, including both more urban and more rural hospitals of varying volumes. Inclusion rates were clearly shown to not depend on case volume or university status, instead, it must be hypothesised that dedicated clinical researchers and their teams are of crucial importance for a high inclusion rate, and thus representativeness. Again, the fact that this assessment of external validity was performed during an ongoing trial offers opportunities to impact on current selection mechanisms through repeated feed-back to the sites.

The broadening of inclusion criteria in February 2016 to allow patients planned for NACT to enter the trial was implemented in a time when the debate of when to perform SLN biopsy in the neoadjuvant setting was still hot. The measure was in compliance with Swedish national guidelines at that time, recommending the use of SLN biopsy before the start of NACT in order to identify node-positive patients that would potentially benefit from adjuvant regional radiotherapy. Since ALND was the recommended axillary intervention in all NACT patients at the time of trial initiation, the introduction of SLN-positive NACT patients into the trial was met with hesitation at first. This may explain the low proportion of NACT patients included into the SENOMAC trial. Today, an overwhelming majority of countries, including Sweden, have abandoned the concept of pre-NACT SLN biopsy. As a consequence, the inclusion of NACT patients into the SENOMAC trial has been suspended. This should not impact negatively on trial results considering the small proportion of such patients having been included.

This analysis may have certain limitations in that it relies on register data as a source for comparison with tightly monitored RCT data. The NKBC, however, has been previously shown to yield acceptable coverage and validity [13], and serves as a unique platform for assessment of external validity.

## Conclusions

This comparative analysis exposes some common issues of external validity in the ongoing SENOMAC trial having today recruited over a third of its target accrual by international collaboration. By presenting this assessment early and not only at conclusion of enrolment, the opportunity is given to impact on selection mechanisms and inclusion rates. Comparisons between study participants and population-based quality registers should be an integral part of practice-changing clinical trials, and data on external validity should always be included in any report of clinical trial outcomes.

## Data Availability

The datasets generated and analysed during the current analysis are not publicly available, as the SENOMAC trial is not yet completed.

## Abbreviations

RCT: Randomised clinical trial
SLN: Sentinel lymph node
NKBC: Swedish National Breast Cancer Register
NACT: Neoadjuvant chemotherapy
ALND: Axillary lymph node dissection
LN: Lymph node
